# Supplementary material for: SMetaS: A Sample Metadata Standardizer for Metabolomics
Source: Metabolites. 2023 Aug 12;13(8):941. doi: 10.3390/metabo13080941 (PMC10456726; doi:10.3390/metabo13080941)
Supplement: Supplementary file 1 [file metabolites-13-00941-s001.zip › metabolites-2527816-supplementary.pdf]

SMetaS: A Sample Metadata Standardizer for Metabolomics

Parker Ladd Bremer [1] and Oliver Fiehn [2,*]

1 Department of Chemistry, University of California, Davis, CA 95616, USA; plbremer@ucdavis.edu
2 West Coast Metabolomics Center for Compound Identification, UC Davis Genome Center, University of California, Davis, CA 95616, USA
* Correspondence: ofiehn@ucdavis.edu

Supplement Material Figure S1

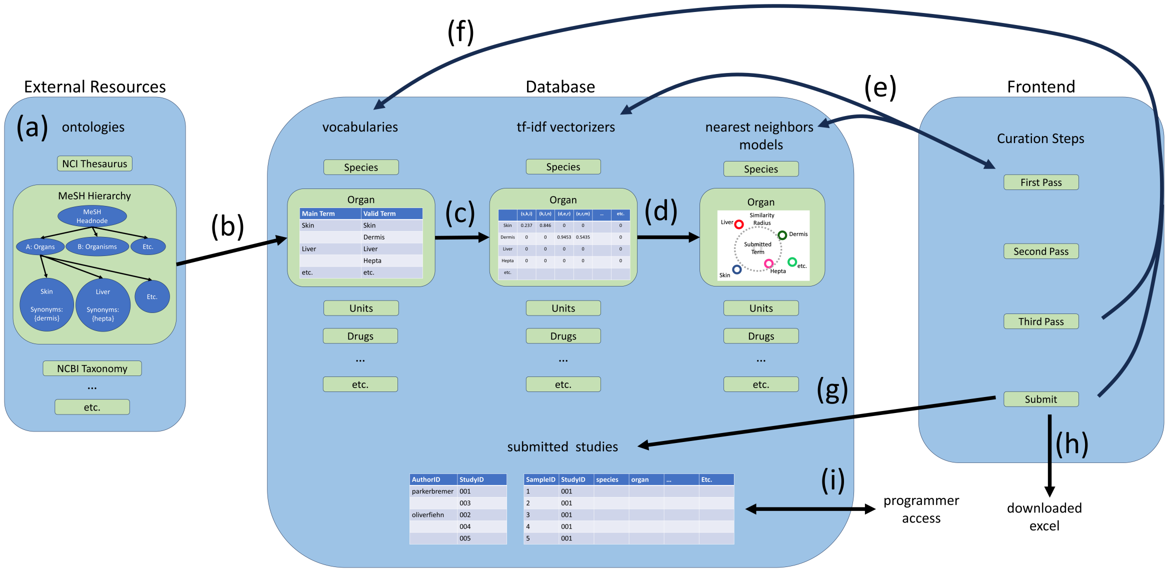

Figure S1: Detailed workflow of SMetaS. Detailed workflow of SMetaS. a) A formal supply of ontologies was curated to relevant terms, with a simplified version of the Medical Subject Headings ontology as example. b) Ontologies are coerced into initial vocabularies for metadata categories. Here, the organ subgraph from MeSH becomes the organ vocabulary. c) each vocabulary generates a tf-idf vectorizer, which creates a numeric space based on triplets of letters. d) each vocabulary and vectorized space is used to create a nearest neighbors model, which is used to find similar terms to strings provided by users. e) in the first pass, described in the Results, the API connects the tf-idf/nearest-neighbors models to the frontend. f) in the event that new terms are added to vocabularies (like a new drug to the drug vocabulary), the API adds terms to the vocabularies, which triggers a retraining of the tf-idf vectorizer/nearest neighbors models based on the expanded vocabulary. g) completion of a curation process stores the resultant curated values into tables in the database. h) completion of a curation process generates an excel file that is immediately available for the user. This excel file contains the same information that is stored in g). i) the stored studies can be pro-grammatically accessed. Details for practical usage are provided in the documentation website at https://metabolomics-us.github.io/metadatastandardizer/.
